# Mutational Dynamics of Aroid Chloroplast Genomes II

**DOI:** 10.3389/fgene.2020.610838

**Published:** 2021-01-20

**Authors:** Abdullah, Claudia L. Henriquez, Thomas B. Croat, Peter Poczai, Ibrar Ahmed

**Affiliations:** ^1^Department of Biochemistry, Faculty of Biological Sciences, Quaid-i-Azam University, Islamabad, Pakistan; ^2^Department of Ecology and Evolutionary Biology, University of California, Los Angeles, Los Angeles, CA, United States; ^3^Missouri Botanical Garden, St. Louis, MO, United States; ^4^Finnish Museum of Natural History, University of Helsinki, Helsinki, Finland; ^5^Alpha Genomics Private Limited, Islamabad, Pakistan

**Keywords:** Araceae (aroid), chloroplast genome, correlations, repeats, InDels (insertions/deletions)

## Abstract

The co-occurrence among single nucleotide polymorphisms (SNPs), insertions-deletions (InDels), and oligonucleotide repeats has been reported in prokaryote, eukaryote, and chloroplast genomes. Correlations among SNPs, InDels, and repeats have been investigated in the plant family Araceae previously using pair-wise sequence alignments of the chloroplast genomes of two morphotypes of one species, *Colocasia esculenta* belonging to subfamily Aroideae (crown group), and four species from the subfamily Lemnoideae, a basal group. The family Araceae is a large family comprising 3,645 species in 144 genera, grouped into eight subfamilies. In the current study, we performed 34 comparisons using 27 species from 7 subfamilies of Araceae to determine correlation coefficients among the mutational events at the family, subfamily, and genus levels. We express strength of the correlations as: negligible or very weak (0.10–0.19), weak (0.20–0.29), moderate (0.30–0.39), strong (0.40–0.69), very strong (0.70–0.99), and perfect (1.00). We observed strong/very strong correlations in most comparisons, whereas a few comparisons showed moderate correlations. The average correlation coefficient was recorded as 0.66 between “SNPs and InDels,” 0.50 between “InDels and repeats,” and 0.42 between “SNPs and repeats.” In qualitative analyses, 95–100% of the repeats at family and sub-family level, while 36–86% of the repeats at genus level comparisons co-occurred with SNPs in the same bins. Our findings show that such correlations among mutational events exist throughout Araceae and support the hypothesis of distribution of oligonucleotide repeats as a proxy for mutational hotspots.

## Introduction

The chloroplast (cp) is a double-membrane bound organelle in plants, which plays an important role in photosynthesis (Daniell et al., 2016). The chloroplast genome originated from prokaryotes (Palmer, 1985). It shows uniparental inheritance, maternal in most angiosperms and paternal in some gymnosperms (Neale and Sederoff, 1989; Avni and Edelman, 1991). Many mutational events occur in the cp genome, including InDels, SNPs, inversions, tandem repeats, and oligonucleotide repeats (Poczai and Hyvönen, 2011; Jheng et al., 2012; Xu et al., 2015; Abdullah et al., 2019; Iram et al., 2019; Sablok et al., 2019). Sufficient polymorphism and uniparental inheritance make the chloroplast genome suitable for phylogenetic inference, resolution of taxonomic discrepancies, population genetics, barcoding, and estimation of time of lineage divergence (Poczai et al., 2011; Ahmed, 2014; Poczai and Hyvönen, 2017; Mehmood et al., 2020c; Shahzadi et al., 2020).

Previously, co-existence of mutations was observed among SNPs, InDels, and repeats in prokaryotic and eukaryotic genomes (Silva and Kondrashov, 2002; Hardison et al., 2003; Tian et al., 2008; Chen et al., 2009; Zhu et al., 2009; McDonald et al., 2011). Three alternate hypotheses were suggested to explain the co-existence of mutations. First, the “regional difference hypothesis” suggests that certain regions are more prone to mutations in comparison to other regions (Silva and Kondrashov, 2002; Hardison et al., 2003). The second, “InDel-induced mutation hypothesis” was suggested based on strong association between InDels and substitutions, which suggested the recruitment of error-prone DNA polymerase at point of InDels is the cause of generation of substitutions (Tian et al., 2008; Yang et al., 2009). The third hypothesis suggests high frequency of oligonucleotide repeats in a region of the genome generates InDels and substitutions (McDonald et al., 2011). To repair DNA damage, the existence of a high number of repeats in a region leads to the recruitment of error-prone DNA polymerases, thus the adjacent sequences replicate with a higher error rate compared to other regions (McDonald et al., 2011). Hence, instead of InDel *per se*, this hypothesis places more importance on “regional difference hypothesis.”

Associations have been reported between SNPs, repeats, InDels, and inversions (Mes et al., 2000; Lockhart et al., 2001; Li J. et al., 2018). The role of repeats in the generation of inversions (Kim and Lee, 2005; Whitlock et al., 2010) and InDels (Kawata et al., 1997) has also been reported. However, these observations were made on the bases of few loci instead of complete chloroplast genomes. The first study of associations among SNPs, InDels, and repeats based on genome-wide analyses of complete chloroplast genomes included five species of Araceae (Ahmed et al., 2012). That study suggests the distribution of oligonucleotide repeats could be used as a proxy for mutational hotspots. Following Ahmed et al. (2012), correlations were studied in two species of genus *Cephalotaxus* Siebold & Zucc. ex Endl. (Yi et al., 2013). However, authors observed very weak correlations between “InDels and SNPs” and “repeats and InDels,” whereas moderate correlation was observed between “substitutions and repeats.” Recently, strong correlations were reported among these mutational events in the species of genus *Dendrobium* Sw. (Li et al., 2020), whereas others have described weak to strong correlations in species of the plant family Malvaceae (Abdullah et al., 2020c,d). Hence, the very thorough study by Abdullah and colleagues reported correlations at the family, subfamily, and genus levels among 19 species belonging to seven subfamilies of Malvaceae (Abdullah et al., 2020c).

**FIGURE 1:**
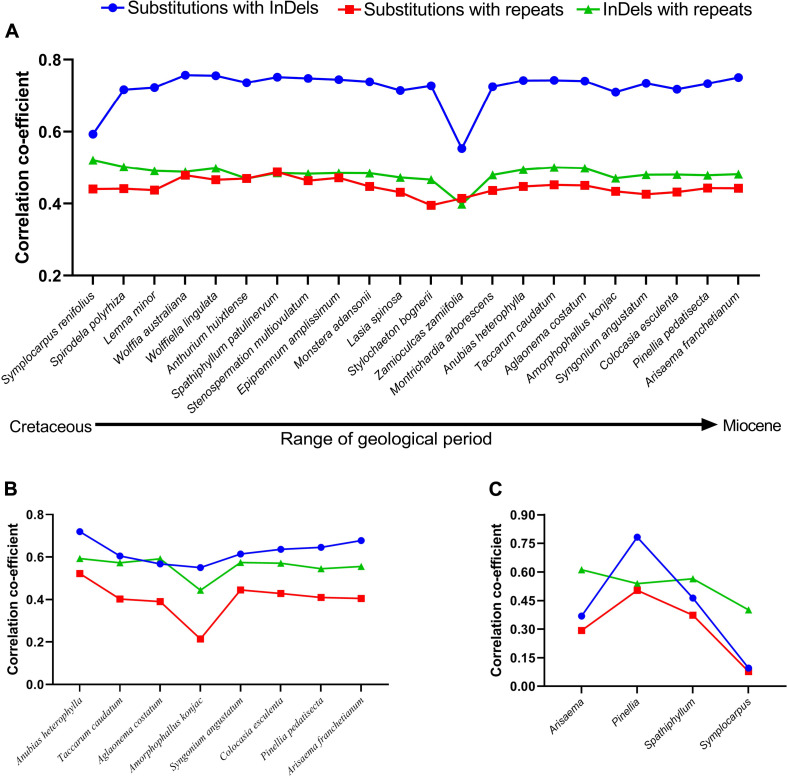
Coefficient of correlations were determined among mutational events using pairwise alignments. **(A)** Family–level comparisons, **(B)** subfamily-level comparisons in Aroideae, **(C)** Genus-level comparisons. *Orontium aquaticum* was used as reference at family level, *Montrichardia arborescence* was used as reference at subfamily level, and at the generic level, *Arisaema franchetianum*, *Pinellia pedatisecta*, *Spathiphyllum patulinervum*, and *Symplocarpus renifolius* were used as references for *Arisaema ringens*, *Pinellia ternata*, *Spathiphyllum kochii*, and *Symplocarpus nipponicus*, respectively.

The previous study of family Araceae was limited to five species of Araceae, including *Colocasia esculenta* (L.) Schott from subfamily Aroideae, which is a younger clade evolutionarily; and four species from subfamily Lemnoideae, which is among the earliest diverging aroid subfamilies (Nauheimer et al., 2012). *Colocasia esculenta* is found in tropical habitat and produces unisexual flowers, whereas the four species of subfamily Lemnoideae produce bisexual flowers and inhabit aquatic habitat (Mayo et al., 1997; Cusimano et al., 2011). These species also demonstrated a different rate of mutations, which is consistent with the finding that aquatic and tropical plant have diverse mutation rates (Abbasi et al., 2016; Hu et al., 2017; Hart et al., 2019; Wang et al., 2020). Sampling is therefore sparse in the previous study for a large and ancient monocot family like Araceae, which dates back to the Early Cretaceous period, and is divided into eight diverse subfamilies distributed across the multitude of ecological habitats (Cusimano et al., 2011; Nauheimer et al., 2012; Henriquez et al., 2014). This family comprises 144 genera and 3,645 species (Boyce and Croat, 2018). Recently, with the advancement of next generation sequencing, chloroplast genome sequences of several species of Araceae were reported from subfamilies Aroideae, Lasioideae, Pothoideae, Monsteroideae, Orontioideae, and Zamioculcadoideae (Han et al., 2016; Choi et al., 2017; Kim et al., 2019; Abdullah et al., 2020a,b; Henriquez et al., 2020a,b). We included 27 species from 7 subfamilies of Araceae which are diverse in term of habit, habitat, native range, and evolutionary time of divergence (Table 1 and Figure 1A). The availability of these genomic resources from a wide array of aroid species (Table 1) provided enough data to elucidate correlations among substitutions, InDels, and repeats throughout the family.

**TABLE 1:** GenBank accession numbers of the species used in comparative analyses along with native range, habit and habitat of each species.

**S. No**	**Species**	**NCBI accession**	**Subfamily**	**Native range**	**Habit and habitat**
1	*Orontium aquaticum*	MT226773	Orontioideae	East United States	Rhizomatous marginal *aquatic* herb grows in ponds, streams, and shallow lakes
2	*Symplocarpus renifolius*	KY039276	Orontioideae	Russian far East to Korea and North & Central Japan	Herb growing on wet places, moist mixed and coniferous forests, forest swamps, swampy meadows and lands
3	*Symplocarpus nipponicus*	MK341566	Orontioideae	Japan, Korea, Manchuria	Herb growing on wet places
4	*Lasia spinosa*	MT226772	Lasioideae	Tropical and subtropical Asia	1–2-m-tall herb. Grow on swamps, riverbanks, ditches, moist places in tropical and subtropical forests, sometimes cultivated along fish ponds and rice fields
5	*Zamioculcas zamiifolia*	MT226775	Zamioculcadoideae	Kenya to KwaZulu-Natal	Tuber subcylindric, ±3–4 cm in diameter or more, tough or woody. Humid to dry evergreen forest, *Brachystegia* woodland, dry wooded grassland, bushland thicket, often on rocks, locally abundant
6	*Stylochaeton bogneri*	MT226774	Zamioculcadoideae	East Tropical Africa	Rhizome slender, horizontal, elongated 0.4–0.6 cm. Evergreen forest, *Brachystegia* woodland
7	*Lemna minor*	DQ400350	Lemnoideae	North and Central America, Temperate and Subtropical Old World	Free-floating aquatic
8	*Spirodela polyrhiza*	JN160603	Lemnoideae	Cosmopolitan	Floating herbs in form of colonies which cover large area of water
9	*Wolffiella lingulata*	JN160604	Lemnoideae	Tropical and Subtropical America	Aquatic herb
10	*Wolffia australiana*	JN160605	Lemnoideae	South and South East Australia, New Zealand	Aquatic herb
11	*Spathiphyllum kochii*	KR270822	Monsteroideae	Colombia to Venezuela	Herb exist on Lowland to middle-elevation forests
12	*Epipremnum aureum*	NC_027954	Monsteroideae	Society Islands (Moorea)	Small herb exists on wet hill forest. ca 500 m
13	*Monstera adansonii*	MN046888	Monsteroideae	South Mexico to Tropical America	Herb, creeper or hemiepiphyte ranged in size from 2 to 4 m climbing on the tree in dense rain forest
14	*Stenospermation multiovulatum*	MN046893	Monsteroideae	West Colombia to Ecuador	Epiphyte herb
15	*Spathiphyllum patulinervum*	MN046890	Monsteroideae	Tropical region of the America	Evergreen herb
16	*Anthurium huixtlense*	MN996266	Pothoideae	Mexico to Central America	Terrestrial or epiphytic, stem ranged to 14 cm long
17	*Pinellia ternata*	KR270823	Aroideae	China to Temperate East Asia	Small herb grows on grasslands, cultivated lands, secondary forests, wastelands,
18	*Colocasia esculenta*	JN105689	Aroideae	India to South China and Sumatera	Robust, acaulescent herb to 2 m. Wild forms occur as colonies on river banks, in open swampy places, on slopes and on rocks and banks in the splash-zone of waterfalls. Very occasionally found in forest under story. Widely cultivated usually near farmhouses or in water fields; also naturalized or perhaps native in wet places in forests, valleys, swamps, wastelands, and at watersides
19	*Arisaema ringens*	MK111107	Aroideae	East China, South Korea, Central Japan to Taiwan	Herbaceous perennial with height of 1–1.5 feet. Grow on humus-rich, moist but well-drained soils in part shade to full shade. Needs consistent moisture and does poorly in heavy clay soils.
20	*Anubias heterophylla*	MN046884	Aroideae	West Central Tropical Africa, Angola	Rhizome creeping, prostrate and rooting, growing on rocky grounds on the banks of or in water courses, and on shady places in the forest
21	*Arisaema franchetianum*	MN046885	Aroideae	South China to North Indo-China	Dioecious plant of up to 1.5 feet, grow in Forests, thickets, and grasslands
22	*Pinellia pedatisecta*	MN046890	Aroideae	Central and South China.	Tuber subglobose, to 4 cm in diam., with some surrounding tubercles. Grow in forests, valleys, shaded areas
23	*Taccarum caudatum*	MN046895	Aroideae	Brazil to Bolivia	Deciduous herbs, grow in rocky area
24	*Montrichardia arborescens*	MN046889	Aroideae	Tropical America	Aquatic herb
25	*Aglaonema costatum*	MN046881	Aroideae	Bangladesh to Peninsula Malaysia	Herb up to 35 cm tall. Grow in dry lowland to hill evergreen forest, mixed evergreen and deciduous forest
26	*Syngonium angustatum*	MN046894	Aroideae	Mexico to Colombia	Climbing herb
27	*Amorphophallus konjac*	MK611803	Aroideae	China	Tuber brown, slightly glossy, depressed globose, to ca. 20 cm high, to ca. 30 cm in diam., seasonally producing numerous long rhizomatous offsets with swollen apical part, these to ca. 50 × 3 cm. Open situations or forest margins and thickets, secondary forests

*The data of distributions, habit and habitat are taken from powo.science.kew.org and Mayo et al. (1997).*

In the current study, we are interested in determining correlations among these mutational events throughout the family Araceae using genus-, subfamily-, and family-level comparisons, *aka* time of divergences ranged from relatively recent splits to deep divergences. This study will be helpful to understand whether such correlations exist among these five species used in Ahmed et al. (2012) by chance or whether these correlations exist among species of Araceae at varying taxonomic levels and diverse ecological habitats.

## Materials and Methods

We downloaded chloroplast genome sequences of 27 species of Araceae from GenBank of the National Center for Biotechnology Information (Table 1). The species are high diverse in terms of habitat, geographical distribution, ecology, and evolutionary history. The species included in the comparisons range in distribution from tropical and subtropical to temperate regions of the world, such as America, Asia, and Africa (Table 1). Similarly, these species also differed in terms of habit and habitat occupying aquatic and semi-aquatic to tropical and subtropical forests (Table 1). The sub-families diverged during Cretaceous to Miocene periods (Nauheimer et al., 2012). We selected one species per genus from all subfamilies other than subfamily Aroideae for family level comparisons. From subfamily Aroideae, we selected 9 species from the comparisons among the major clades using a previous phylogenetic inference of Araceae (Cusimano et al., 2011; Henriquez et al., 2014). We performed comparisons at the family, subfamily, and genus levels. At the family level, all the species were pairwise compared with *Orontium aquaticum* L. (Orontioideae) which is among the basal groups of Araceae following a previous approach applied in family Malvaceae (Abdullah et al., 2020c). At the subfamily level in Aroideae, the genome of *Montrichardia arborescens* (L.) Schott is used as a reference for the other species of subfamily Aroideae. At the generic level, *Arisaema franchetianum* Engler, *Pinellia pedatisecta* Schott, *Spathiphyllum patulinervum* G. S. Bunting, and *Symplocarpus renifolius* Schott ex Tzvelev were used as references for *Arisaema ringens* (Thunb.) Schott, *Pinellia ternata* (Thunb.) Makino, *Spathiphyllum kochii* Engl. & K. Krause, and *Symplocarpus nipponicus* Makino, respectively.

The MAFFT (Multiple alignment using fast Fourier transform) integrated in Geneious R8.1 (Kearse et al., 2012) was used for the pairwise alignment in all comparisons after removal of long inverted repeat regions following Ahmed et al. (2012). We also deleted *ycf*1 and *rps*15 genes along with intergenic-spacer regions, as these genes jump between small single-copy and inverted-repeat regions, hence present the problem of rate heterotachy (Lockhart et al., 2006; Abdullah et al., 2020a). Each alignment was divided into non-overlapping bins of 250 bp and deletions in the reference genome were removed from the alignment after noting their positions. This approach has been used previously (Ahmed et al., 2012; Yi et al., 2013; Abdullah et al., 2020c) to fix the coordinates positions in the reference genomes for allocations of oligonucleotide repeats. The InDels were counted manually and assigned into bins of 250 bp. The forward and reverse repeats were determined as ≥ 14 bp using REPuter (Kurtz et al., 2001) by searching for 5,000 repeats in the reference genomes at family, subfamily, and generic levels. The names of the species whose cp genomes were used as reference are mentioned above (*vide infra*). All the repeats with exact match located at least 10 bp away from each other were included in the analyses after excluding redundant repeats. The repeats were allocated into bins using Microsoft Excel (Redmond, United States). The numbers of substitutions were determined by a custom Pearl script and allocated into bins in Microsoft Excel.

Quantitative and qualitative approaches were used to determine the correlations among the mutational events. The normality test was first performed on the data in Minitab v.19 following Abdullah et al. (2020c). This test confirmed the non-normal distribution of mutational events (Supplementary Figures S1–S4). Hence, Spearman *rho* (ρ) correlations were applied on the non-normal data in Minitab v.19. The methodology described in Akoglu (2018) was used to express strength of the correlations as follows: negligible or very weak (0.10–0.19), weak (0.20–0.29), moderate (0.30–0.39), strong (0.40–0.69), very strong (0.70–0.99), and perfect (1.00). The probability (*p*) of significance of correlations was determined at 0.05 α level.

In the qualitative approach, we evaluated the co-occurrence of InDels with substitutions, and of repeats with InDels and substitutions following Abdullah et al. (2020c).

## Results

### Correlations Among SNPs, InDels, and Oligonucleotide Repeats at the Family Level

Among 22 comparisons at the family level, the correlations between SNPs and InDels were strong for *Symplocarpus renifolius* and *Zamioculcas zamiifolia* (Lodd.) Engl., whereas were categorized as very strong in the remaining 20 comparisons (Figure 1A). Correlations between SNPs and repeats were regarded as strong for all other comparisons except *Stylochaeton bogneri* Mayo, which showed moderate correlations (Figure 1A). We recorded strong correlations between repeats and InDels in all comparisons. The average values of coefficients of correlations were recorded highest between substitutions and InDels (0.72), followed by InDels and repeats (0.48), and then by substitutions and repeats (0.44). All correlations were observed with a high significance of *p* < 0.0001. All the comparisons showed high similarities in correlations from basal groups to the crown group. The distributions of substitutions, InDels, and repeats in 250 bp bins are shown in Supplementary Table S1.

### Correlations Among SNPs, InDels, and Oligonucleotide Repeats at the Subfamily Level

For eight comparisons within the subfamily Aroideae, strong correlations were observed among SNPs and InDels for seven comparisons, whereas a very strong correlation was observed for *Anubias heterophylla* Engl. (Figure 1B). We recorded strong correlations between SNPs and repeats for six comparisons, whilst moderate correlation was recorded for *Aglaonema costatum* N.E.Br., and weak correlation was recorded in *Amorphophallus konjac* K. Koch (Figure 1B). We observed strong correlations between InDels and repeats for all comparisons (Figure 1B). The average values of correlation coefficients showed a similar pattern as observed at the family-level comparisons: it remained highest between substitutions and InDels (0.62), followed by InDels and repeats (0.55), and then by substitutions and repeats (0.40). All correlations at the subfamily level were also observed with high significance of *p* < 0.0001. The distributions of substitutions, InDels, and repeats in 250 bp bins are shown in Supplementary Table S2.

### Correlations Among SNPs, InDels, and Oligonucleotide Repeats at the Genus Level

We investigated interspecific correlations in four genera as representative of recent splits between species belonging to the same genera. The correlation coefficients greatly varied in these comparisons; the correlations between SNPs and InDels remained very strong between the species of genus *Pinellia* Ten., strong in *Spathiphyllum* Schott, moderate in *Arisaema* Mart., and negligible in *Symplocarpus* Salisb. (Figure 1C). The same pattern was evident for correlations between substitutions and repeats, which remained strong in *Pinellia*, moderate in *Spathiphyllum*, weak in *Arisaema*, and negligible in *Symplocarpus* (Figure 1C). Conversely, all comparisons showed strong correlations between repeats and InDels (Figure 1C). In these comparisons, the average values of the coefficients of correlations were found highest between repeats and InDels (0.52), followed by SNPs and InDels (0.42), and SNPs and repeats (0.31). Except *Symplocarpus*, correlations in all other comparisons were observed with *p* < 0.0001. Low significance was observed for substitutions and InDels (*p* = 0.024), and for substitutions and repeats (*p* = 0.055) in *Symplocarpus*. The distributions of substitutions, InDels, and repeats in 250 bp bins are shown in Supplementary Table S3.

### Qualitative Analyses of the Existence of InDels With Substitutions, and of Repeats With Substitutions and InDels

In the qualitative analyses, we determined the percentages of the InDel-containing bins that co-occurred with SNPs, and of the repeat-containing bins that co-occurred with InDels and SNPs. At the family level, we observed that up to 99.47–100% of InDel-containing bins also contained SNPs, 97.88–100% of repeat-containing bins also showed SNPs, and up to 66.45–80.51% of repeat-containing bins also contained InDels (Table 2).

**TABLE 2:** The co-occurrence of InDels with substitutions, and of repeats with substitutions and InDels in family Araceae.

**Species**	**SNPs with InDels (%)**	**InDels with repeats (%)**	**SNPs with repeats (%)**
**Family level**			
*Aglaonema costatum*	100	76.69	99.57
*Amorphophallus konjac*	100	75.00	100
*Anthurium huixtlense*	100	77.11	99.57
*Anubias heterophylla*	100	75.00	99.57
*Arisaema franchetianum*	100	75.85	100
*Colocasia esculenta*	100	76.27	100
*Epipremnum amplissimum*	100	77.00	100
*Lasia spinosa*	100	76.69	99.57
*Lemna minor*	100	78.72	100
*Monstera adansonii*	100	77.11	100
*Montrichardia arborescens*	100	74.57	99.57
*Pinellia pedatisecta*	100	75.00	99.57
*Spathiphyllum patulinervum*	100	76.69	100
*Spirodela polyrhiza*	100	78.39	100
*Stenospermation multiovulatum*	100	76.69	100
*Stylochaeton bogneri*	100	76.69	97.88
*Symplocarpus renifolius*	99.47	64.45	99.57
*Syngonium angustatum*	100	74.58	99.57
*Taccarum caudatum*	100	76.27	99.57
*Wolffia australiana*	100	80.51	100
*Wolffiella lingulata*	100	80.10	100
*Zamioculcas zamiifolia*	99.63	80.50	99.57
**Subfamily level**			
*Aglaonema costatum*	100	66.00	98.66
*Amorphophallus konjac*	99.57	63.75	94.95
*Anubias heterophylla*	97.98	60.73	97.98
*Arisaema franchetianum*	100	79.80	98.99
*Colocasia esculenta*	100	67.11	100
*Pinellia pedatisecta*	99.57	66.10	98.66
*Syngonium angustatum*	100	64.76	99.33
*Taccarum caudatum*	100	66.10	99.33
**Genus level**			
*Arisaema*	85.71	75.16	81.36
*Pinellia*	71.08	42.66	36.00
*Spathiphyllum*	90.55	64.18	86.51
*Symplocarpus*	23.73	20.28	15.66

The results at the subfamily level show high similarities with the family level. We observed 97.98–100% of InDel-containing bins that also contained SNPs, 94.95–100% of repeat-containing bins also contained SNPs, whereas up to 60.73–80% of repeat-containing bins also exhibited InDels (Table 2). In genus-level comparisons, for qualitative comparisons of three among the four genera, 71.08–90.55% of InDel-containing bins exhibited SNPs, 42.66–75.16% of repeat-containing bins also contained InDels, while 36–86.51% of the repeat-containing bins also displayed SNPs. The genus *Symplocarpus* remained an exception, for which only 23.73% of InDel-containing bins showed SNPs, and only 20.28% of repeat-containing bins exhibited InDels, while merely 15.66% of repeat-containing bins displayed SNPs (Table 2).

### Distributions of InDels and Substitutions at Family, Subfamily, and Genus Level

At the family level, the distantly related species showed existence of a high number of substitutions and InDels with 3,430–15,459 substitutions and 456–1,156 InDels. Most of the substitutions and InDels were found in aquatic species of subfamily Lemnoideae (Table 3). At the subfamily level, deeply diverge species showed 3,639–5,859 substitutions and 537–765 InDels. At the genus level, 89–1,793 substitutions and 70–352 InDels were determined in closely related species (Table 3). The species of genus *Symplocarpus* show a low number of substitutions and InDels 89 and 70, respectively.

**TABLE 3:** Distribution of SNPs and InDels at family, subfamily, and genus level.

**Species**	**SNPs**	**InDels**
**Family level**		
*Aglaonema costatum*	9,283	991
*Amorphophallus konjac*	9,849	956
*Anthurium huixtlense*	10,533	1,007
*Anubias heterophylla*	9,283	957
*Arisaema franchetianum*	10,343	1,006
*Colocasia esculenta*	9,819	950
*Epipremnum amplissimum*	9,826	989
*Lasia spinosa*	10,193	1,019
*Lemna minor*	13,424	1,060
*Monstera adansonii*	9,736	979
*Montrichardia arborescens*	9,248	922
*Pinellia pedatisecta*	10,190	964
*Spathiphyllum patulinervum*	10,003	971
*Spirodela polyrhiza*	11,624	1003
*Stenospermation multiovulatum*	9,701	959
*Stylochaeton bogneri*	9,783	1,005
*Symplocarpus renifolius*	3,430	456
*Syngonium angustatum*	9,682	977
*Taccarum caudatum*	9,712	964
*Wolffia australiana*	15,459	1,147
*Wolffiella lingulata*	15,238	1,156
*Zamioculcas zamiifolia*	9,336	958
**Subfamily Aroideae level**		
*Aglaonema costatum*	3,639	571
*Amorphophallus konjac*	3,750	549
*Anubias heterophylla*	5,859	537
*Arisaema franchetianum*	5,592	765
*Colocasia esculenta*	4,704	638
*Pinellia pedatisecta*	5,161	707
*Syngonium angustatum*	4,308	620
*Taccarum caudatum*	4,061	628
**Genus level**		
*Arisaema ringens vs. Arisaema franchetianum*	1,355	303
*Pinellia ternata vs. Pinellia pedatisecta*	1,793	173
*Spathiphyllum kochii vs. Spathiphyllum patulinervum*	1,662	352
*Symplocarpus nipponicus* VS *Symplocarpus renifolius*	89	70

## Discussion

We determined the extent of correlations among SNPs, InDels, and repeats in cp genomes using 27 species from 23 genera, distributed among seven of the eight subfamilies of Araceae. We performed 34 pairwise comparisons and observed strong/very-strong correlations for most of the comparisons among these mutational events, which suggests high associations between these mutational events.

We removed the *ycf*1 and *rps*15 genes, along with intergenic spacer regions, as these elements are located at the single-copy and inverted-repeat junctions—appearing in single-copy regions in some species, and in inverted repeats regions in others. Single-copy regions undergo a different rate of mutation compared to the inverted-repeat regions, hence the same genes that occur in single-copy regions in some species and in inverted-repeats in other species undergo a phenomenon known as rate heterotachy (Lockhart et al., 2006). We previously reported the effect of rate heterotachy in Araceae (Abdullah et al., 2020a). Single nucleotide polymorphisms, InDels, and oligonucleotide repeats did not follow the normal distribution curves in normality tests using Minitab v.19. These observations are in agreement with previous reports of chloroplast genomes in which certain regions were found to be predisposed to mutations and reported as hotspots for mutations (Ahmed et al., 2013; Li Y. et al., 2018; Sablok et al., 2019; Abdullah et al., 2020e; Mehmood et al., 2020a,b).

Ahmed et al. (2012) determined correlations among SNPs, InDels, and repeats using chloroplast genomes of two morphotypes of one species, *C. esculenta*, and four species of the subfamily Lemnoideae, including *Lemna minor* L., *Wolffia australiana* (Benth.) Hartlog & Plas, *Wolffiella lingulata* Hegelm., and *Spirodela polyrhiza* (L.) Schleid. *Colocasia esculenta* is tropical and belongs to the crown group, whereas the species of Lemnoideae are aquatic and belong to the basal group. Aquatic plants evolve faster as compared to non-aquatic, and tropical plants evolve faster as compared to temperate plants (Abbasi et al., 2016; Hu et al., 2017; Hart et al., 2019; Wang et al., 2020). We found higher rates of mutation in terms of substitutions and InDels in the species of Lemnoideae as compared to other species (Table 3). Hence, further exploration of these observations was required in diverse species to gain insight into correlations among mutational events as sparse sampling of taxa is evident in the previous study of Ahmed et al. (2012). In order to cover the taxa across the family tree, here we include species spanning seven of the eight subfamilies of Araceae and used 34 comparisons among 27 diverse species in terms of habit, habitat, and evolution.

At the family and subfamily levels, most of the comparisons exhibited strong/very strong correlations among “SNPs and InDels,” “SNPs and repeats,” and “InDels and repeats.” Hence, our study confirms strong correlations among mutational events in close comparisons (subfamily level) and distant comparisons (family level). Here, the high similarity among mutational events in diverse species in terms of geography, ecology, and time of divergence (Table 1 and Figure 1A) demonstrates that the correlations are unaffected by the geographical distribution, habit, and habitat. Weak correlations in generic-level comparisons, however, may be due to fewer SNPs and InDels in recently diverged species within the same genera. Strong correlations have also been reported in the family Malvaceae (Abdullah et al., 2020c). At the genus level, we observed very weak to strong correlations among mutational events. Similar results were reported in the family Malvaceae at the genus level (Abdullah et al., 2020c). Here, very weak correlations were recorded between the species of *Symplocarpus*. The species of *Symplocarpus* showed closed resemblance and revealed the presence of few substitutions (89) and InDels (70). Hence, the weak correlations might be due to recent divergence of these species from each other. Similar results were observed in the closely related species of *Theobroma* L. (Abdullah et al., 2020c) and *Cephalotaxus* (Yi et al., 2013). Previously, Tian et al. (2008) suggested InDels as mutagens, whereas McDonald et al. (2011) suggested the role of repeats in the generation of InDels and SNPs. However, they considered the recruitment of error-prone DNA polymerases during replication to be the cause of high mutations due to errors in replications. Therefore, in closely related species InDels and repeats might not have enough time to generate substitutions. Moreover, correlations between “InDels and repeats” were found to be higher than correlations between “SNPs and InDels” and “SNPs and repeats” in three out of four comparisons. Similar results were previously observed in family Malvaceae, where four of the five comparisons showed high correlation between “InDels and repeats” as compared to “SNPs and InDels” and “SNPs and repeats” (Abdullah et al., 2020c). These observations at the genus level suggest that most of the InDels are generated by repeats first, and then both InDels and repeats contribute to the generation of SNPs over a period of time.

The quantitative analyses showed very strong correlations between SNPs and InDels in most cases, whereas the qualitative analyses revealed the occurrence of more than 90% of InDels containing bins with SNPs. Previously strong associations were also observed among SNPs and InDels in prokaryotic, eukaryotic, and chloroplast genomes (Tian et al., 2008; Zhang et al., 2008; Chen et al., 2009; Yang et al., 2009; Abdullah et al., 2020c; Li et al., 2020). The InDels were suggested as a mutagen for the generation of SNPs based on the observation of high association between InDels and SNPs in prokaryotic and eukaryotic genomes (Tian et al., 2008; Zhu et al., 2009). Our analyses lend support to these previous results. Chloroplast genomes originate from prokaryotes and decrease in size by loss of genomic portions along with several genes (Palmer, 1985) but still reveal high associations between SNPs and InDels.

Abdullah et al. (2020c) reported weak to moderate correlations between “SNPs and repeats” and “InDels and repeats” in most of the comparisons in the plant family Malvaceae. However, based on qualitative analyses, they reported the existence of up to 60% of repeats with InDels and up to 90% of repeats with SNPs. In the current study, we report strong correlations between “InDels and repeats” and “SNPs and repeats” based on quantitative analyses in the family Araceae, whereas based on qualitative analyses we observed the existence of up to 100% of repeats with SNPs and up to 80% of repeats with InDels. The variation in the results might be due to the inclusion of a copy of inverted repeats in comparisons of family Malvaceae as the inverted-repeats region showed less polymorphism due to copy-dependent repair mechanisms (Zhu et al., 2016). Here we excluded one copy of the Inverted repeats from our comparisons, following previous studies (Ahmed et al., 2012; Yi et al., 2013). A high frequency of repeats has previously been considered the cause of generations of SNPs and InDels in the adjacent regions based on strong associations between “InDels and repeats” and “SNPs and repeats” in prokaryotic and eukaryotic genomes (McDonald et al., 2011). Here, our analyses in a wider sampling of species of Araceae and the previous report of Malvaceae (Abdullah et al., 2020c) also support the role of repeats in the generation of InDels and substitutions, and supports the hypothesis that oligonucleotide repeats can be used as a proxy for identification of mutational hotspots (Ahmed et al., 2012; Abdullah et al., 2020c). This hypothesis has practical implications in selecting appropriate loci for comparative analyses. No one single locus is good enough for evolutionary comparisons at all time scales; slow evolving regions should be preferred for deep divergences, while mutational hotspots for the closely related taxa and recently diverged species (Ahmed et al., 2013; Ahmed, 2015; Li et al., 2020). A recent report of Ahmed et al. (2020) on family Araceae showed the practical implication of the use of repeats in identification of suitable polymorphic loci for the study of phylogeography and population genetics. Their developed markers from the identified loci providing new insight about the origin of *Colocasia esculenta* in southeast Asia instead of Papua New Guinea (Ahmed et al., 2020). Our current results support strong associations between repeats and substitutions and repeats and InDels in Araceae, which can be helpful for identifying species-specific suitable loci for the study of phylogeography, domestication, and population genetics of other species of Araceae.

In conclusion, the previous observations in five aroid species were not an artifact of low sampling but a representative sample of the correlations found at various taxonomic levels, and in ecologically, geographically and evolutionarily of Araceae. The strong associations of InDels with SNPs, and of repeats within InDels and SNPs, support the previous observation (Ahmed et al., 2012) that the multiple hypotheses outlined in the introduction (*vide infra*) might explain the mutational dynamics of chloroplast genome evolution. The strong associations among the three types of mutational events reported in prokaryotic, eukaryotic (Tian et al., 2008; Zhang et al., 2008; Chen et al., 2009; McDonald et al., 2011), and chloroplast genomes (Ahmed et al., 2012; Abdullah et al., 2020c; Li et al., 2020), show that such co-occurrence of mutations might be a universal phenomenon in all types of genomes. Further studies in prokaryotes and eukaryotes are needed to test this hypothesis.

## Data Availability Statement

Publicly available datasets were analyzed in this study. This data can be found here: All the accession numbers are given in Table 1. Moreover, result of analyses are also provided in the article or as Supplementary Material.

## Author Contributions

A: data analyses, data interpretation, writing initial draft, and conceptualization. CH: data analyses, review and editing of initial draft. TC: data interpretation and conceptualization. PP and IA: conceptualization, review, editing of initial draft, and supervision. All authors contributed to the article and approved the submitted version.

## Conflict of Interest

IA was employed by company Alpha Genomics Private Limited, Islamabad, Pakistan. The remaining authors declare that the research was conducted in the absence of any commercial or financial relationships that could be construed as a potential conflict of interest.

## Supplementary Material

Supplementary Figure 1 Represent the non-normal distribution of SNPs, InDels, and repeats in *Wolffia australiana.*

Supplementary Figure 2 Represent the non-normal distribution of SNPs, InDels, and repeats in *Anthurium huixtlense.*

Supplementary Figure 3 Represent the non-normal distribution of SNPs, InDels, and repeats in *Taccarum caudatum.*

Supplementary Figure 4 Represent the non-normal distribution of SNPs, InDels, and repeats in *Aglaonema costatum*.

Supplementary Table 1 Distributions of substitutions, InDels, and oligonucleotide repeats in bins of 250 bp in pairwise comparisons at family level.

Supplementary Table 2 Distributions of substitutions, InDels, and oligonucleotide repeats in bins of 250 bp in pairwise comparisons at subfamily level.

Supplementary Table 3 Distributions of substitutions, InDels, and oligonucleotide repeats in bins of 250 bp in pairwise comparisons at genus level.
