# Supplementary figures and images for: Mutational Dynamics of Aroid Chloroplast Genomes II
Source: Front Genet. 2021 Jan 20;11:610838. doi: 10.3389/fgene.2020.610838 (PMC7854696; doi:10.3389/fgene.2020.610838)

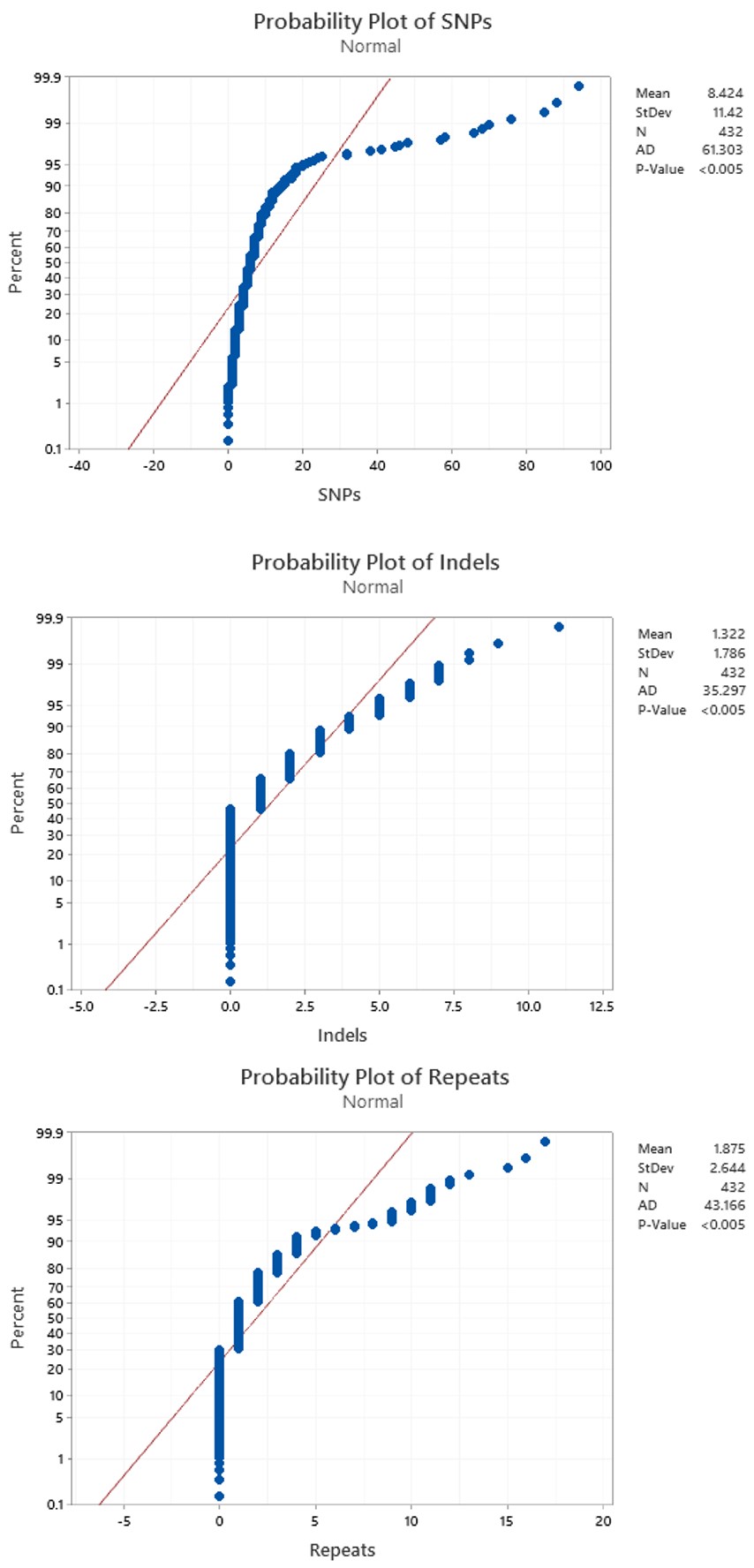

Supplement: Supplementary Figure 1 — Represent the non-normal distribution of SNPs, InDels, and repeats in Wolffia australiana. [file Image_1.JPEG]

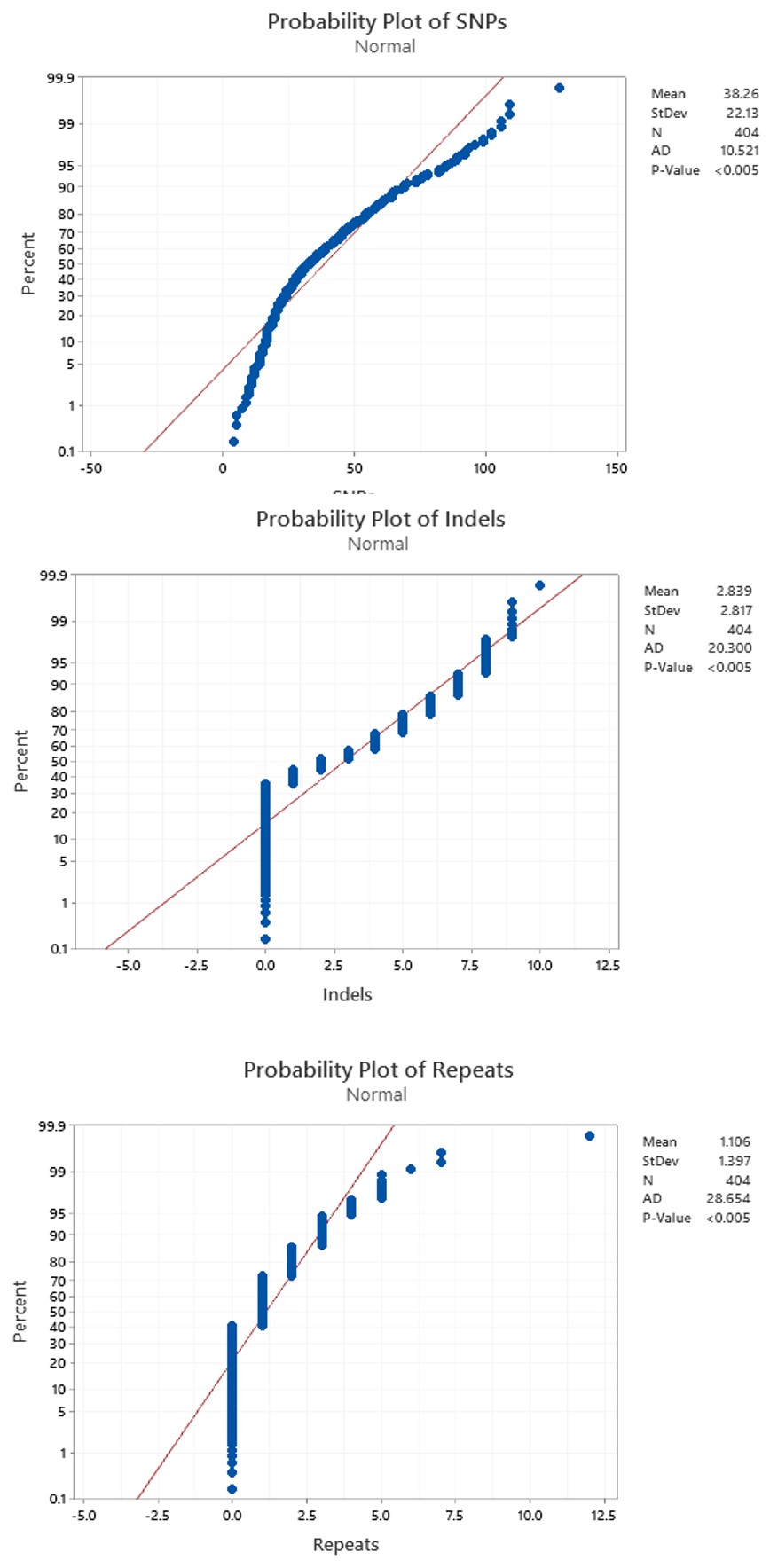

Supplement: Supplementary Figure 2 — Represent the non-normal distribution of SNPs, InDels, and repeats in Anthurium huixtlense. [file Image_2.JPEG]

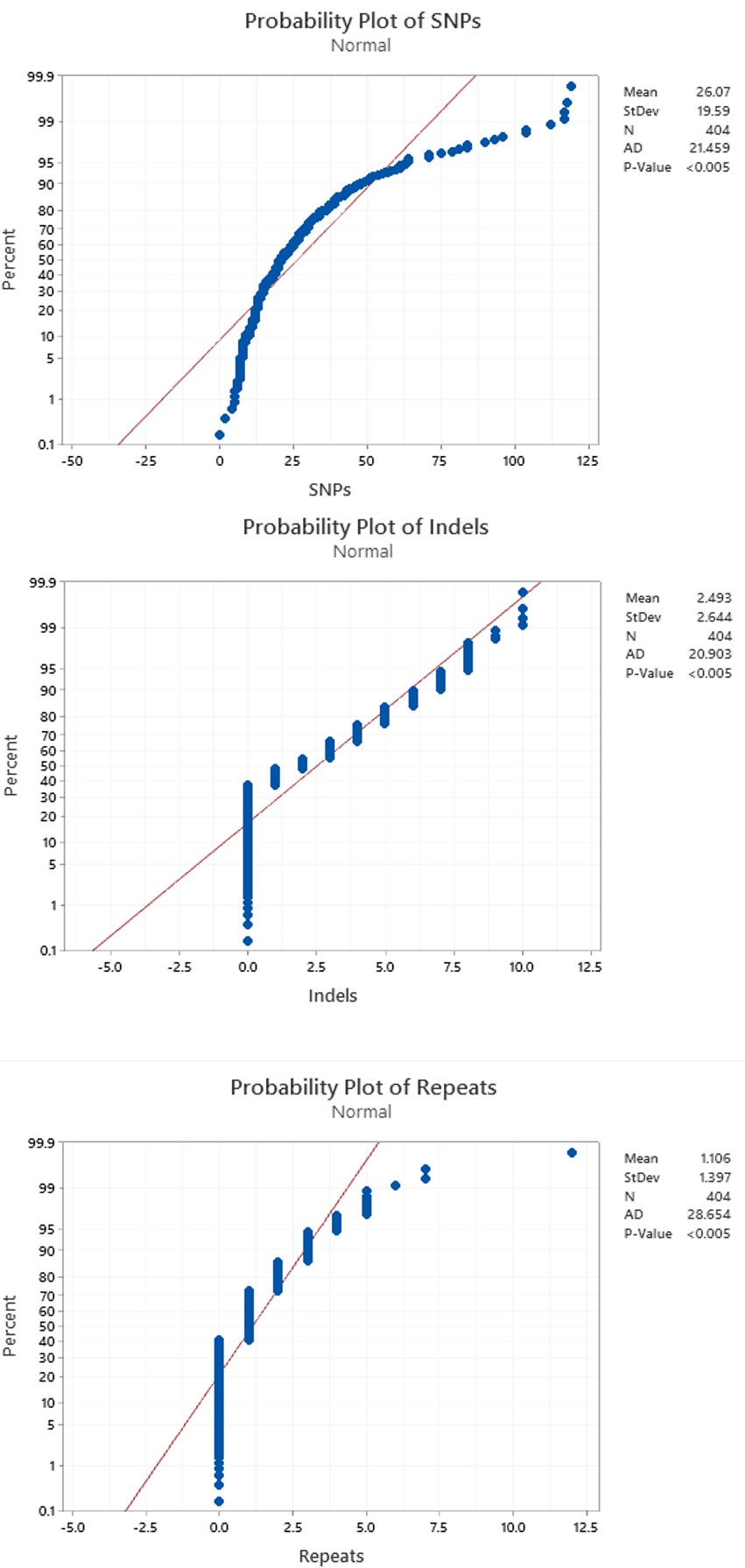

Supplement: Supplementary Figure 3 — Represent the non-normal distribution of SNPs, InDels, and repeats in Taccarum caudatum. [file Image_3.JPEG]

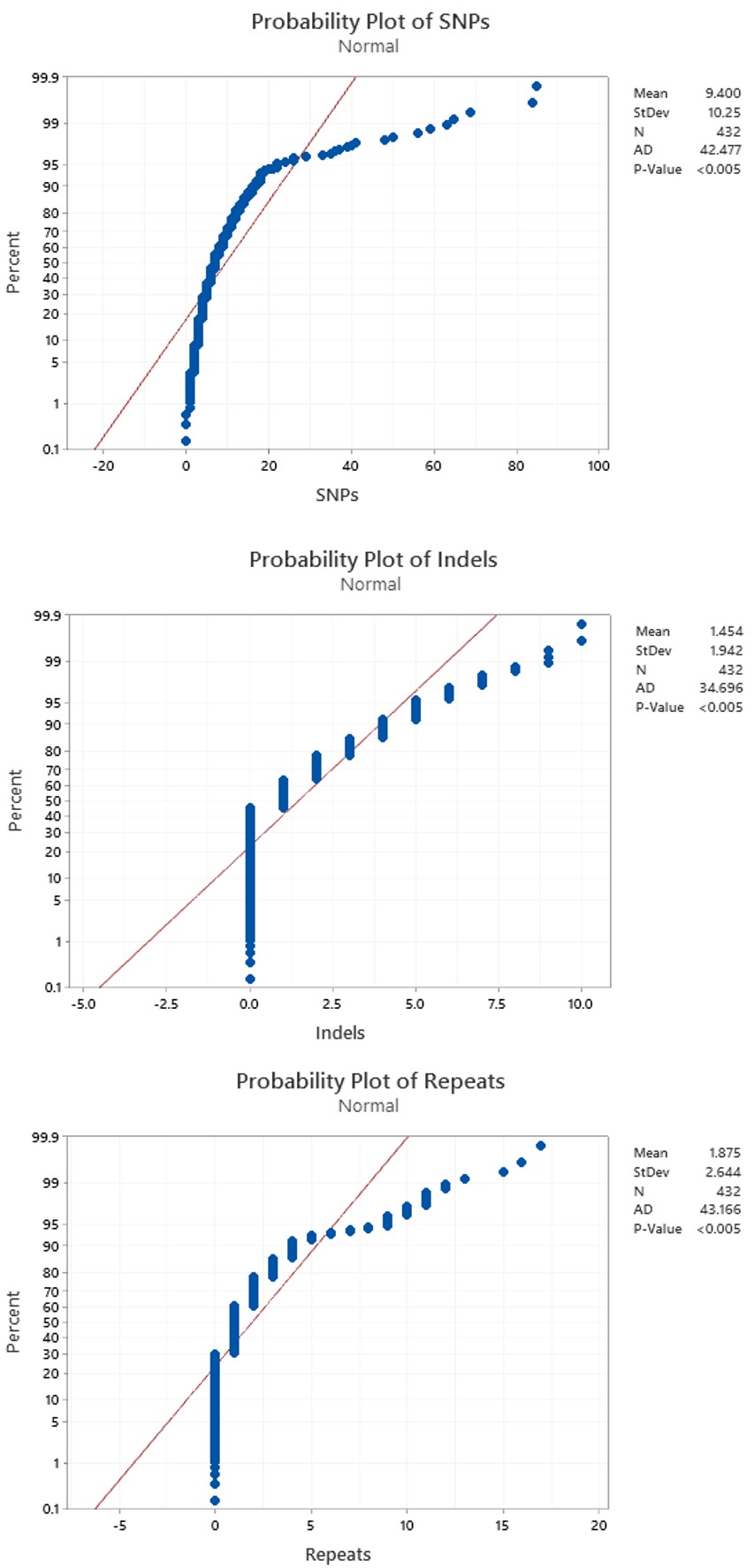

Supplement: Supplementary Figure 4 — Represent the non-normal distribution of SNPs, InDels, and repeats in Aglaonema costatum. [file Image_4.JPEG]
